# Management and Treatment Outcomes of Hemolytic Disease of the Fetus and Newborn (HDFN)—A Retrospective Cohort Study

**DOI:** 10.3390/jcm13164785

**Published:** 2024-08-14

**Authors:** Agnieszka Drozdowska-Szymczak, Sabina Łukawska, Natalia Mazanowska, Artur Ludwin, Paweł Krajewski

**Affiliations:** 1 Department of Neonatology and Neonatal Intensive Care, Institute of Mother and Child, Kasprzaka 17a, 01-211 Warsaw, Poland; agnieszka.drozdowska@imid.med.pl (A.D.-S.); pawel.krajewski@imid.med.pl (P.K.); 2 Department of Obstetrics and Gynecology, Institute of Mother and Child, Kasprzaka 17a, 01-211 Warsaw, Poland; natalia.mazanowska@imid.med.pl; 3 Department of Obstetrics and Gynecology, Medical University of Warsaw, Pl. Starynkiewicza 1/3, 02-015 Warsaw, Poland; ludwin@cm-uj.krakow.pl

**Keywords:** neonate, newborn, hemolytic disease of the fetus and newborn, HDFN, alloimmunization, hyperbilirubinemia, intrauterine blood transfusion, exchange transfusion, phototherapy

## Abstract

**Background:** Hemolytic disease of the fetus and newborn (HDFN) is caused by maternal antibodies attacking fetal blood cell antigens. Despite routine antenatal anti-D prophylaxis, intrauterine transfusions (IUTs) are still needed in some HDFN cases. **Methods:** We conducted a retrospective cohort study on newborns with HDFN born in the 1st Department of Obstetrics and Gynecology of the Medical University of Warsaw. We analyzed 274 neonates with HDFN, identifying 46 who required IUT due to fetal anemia and 228 who did not. The laboratory results, management, and outcomes were compared between these groups. **Results:** Comparative analysis showed that newborns treated with IUT were more likely to have significant anemia, hyperbilirubinemia, and iron overload, indicated by a high ferritin concentration. These neonates more often required top-up transfusions, phototherapy, intravenous immunoglobulin infusions, and exchange transfusions. The length of stay was longer for newborns who received IUT. **Conclusions:** HDFN requiring IUT is associated with a greater number of complications in the neonatal period and more often requires additional treatment compared to HDFN not requiring IUT.

## 1. Introduction

Hemolytic disease of the fetus and newborn (HDFN) is caused by maternal antibodies directed against Rh, AB0, Kidd, Kell, MNS, Duffy, Lewis, and other blood group antigens present on fetal blood cells. Despite the performance of screening and anti-D prophylaxis in RhD-negative pregnant women, HDFN is still a relevant issue, and many newborns require special treatment and careful observation due to HDFN.

HDFN may manifest differently depending on the severity of symptoms, from mild anemia and jaundice observed after birth, to severe fetal anemia often requiring intrauterine transfusion (IUT), sometimes complicated by hydrops fetalis or even intrauterine fetal demise. IUT procedure carries the risk of complications such as preterm birth, the premature rupture of membranes (PROM), intrauterine infection, as well as fetal circulatory failure resulting from volume overload [1].

The decision to perform IUT is based on the multiple of the median (MoM) for the peak systolic velocity (PSV) in the fetal middle cerebral artery (MCA), as measured during an ultrasound examination. The indication for IUT is a MoM value exceeding 1.5 [2].

Our study aimed to analyze the laboratory test results and management of newborns born from pregnancies complicated by HDFN, as well as to compare the results obtained in neonates who received IUT and those who did not require this procedure.

## 2. Materials and Methods

We conducted an observational, cohort, retrospective study submitted to the Bioethics Committee of the Medical University of Warsaw (AKBE/261/2022). From 1 January 2017 to 31 December 2020, 8340 newborns were hospitalized in the 1st Department of Obstetrics and Gynecology of the Medical University of Warsaw. A review of the electronic database found a total of 274 newborns with a diagnosis of HDFN.

At that time, the 1st Department of Obstetrics and Gynecology was a tertiary center specializing in pregnancies complicated by HDFN. Therefore, the birth rate of children born from such pregnancies in this department is higher than the average incidence in the general population (3.3% vs. 3/100,000 to 80/100,000) [3]. Additionally, our center has a higher percentage of pregnancies complicated by HDFN requiring IUTs (26.7% vs. 13% in the case of Rh and Kell blood group conflicts) [4].

All medical histories of children born between 1 January 2017 to 31 December 2020 from pregnancies complicated by HDFN were analyzed. The inclusion criteria were a positive direct antiglobulin test (DAT) or the performance of IUT due to fetal anemia in the course of HDFN, as in these children, the blood type and DAT were not determined after birth due to the performed IUT. Out of the 274 neonates who qualified for the study, 46 patients required IUT. The decision to perform IUT in these patients was based on the MCA PSV value measured during an ultrasound examination. The MCA PSV, an effective non-invasive tool for assessing the severity of fetal anemia, is measured according to principles that ensure high repeatability. The first ultrasound examination with MCA PSV assessment is typically performed around the 17th to 18th week of pregnancy, and in exceptional cases, earlier if there is a history of early and severe maternal–fetal immunization. In cases of low antibody titers, follow-up examinations are performed every four weeks. For high antibody titers (1:16; and 1:4 in the presence of anti-Kell antibodies), follow-up is performed every two weeks. Patients with an unfavorable history and high antibody titers have follow-up visits scheduled once a week, or more frequently in the case of borderline MCA PSV values. An MCA PSV above 1.5 times the median for the given gestational age indicates fetal anemia and is an indication for performing diagnostic–therapeutic cordocentesis: measuring the hemoglobin concentration in a sample of fetal blood taken from the umbilical vein and, upon confirmation of anemia, simultaneously performing an intrauterine transfusion. The timing of the next transfusion depends on the degree of fetal anemia, the fetal hemoglobin concentration after the transfusion, and the anticipated rate of hemoglobin decline following the procedure [5,6].

Initially, we assessed the baseline characteristics of the study group, including their gender, gestational age, birth weight, and types of red cell alloimmunization. To identify small for gestational age (SGA) neonates, we used the Fenton Growth Charts [7].

In all newborns born from pregnancies complicated by HDFN, the total bilirubin concentration was measured and a blood count was performed in the cord blood sample or right after birth. In patients diagnosed after birth, these tests were performed immediately after establishing the diagnosis.

In the following days, the total bilirubin concentration and blood count were checked several times, depending on the severity of the child’s symptoms. Furthermore, the direct bilirubin concentration was measured at least once, and follow-up was planned based on the obtained results. Also, the serum ferritin concentration was measured in newborns with severe hyperbilirubinemia or anemia. The blood samples were tested by qualified laboratory technicians immediately after sampling in the hospital laboratory. Our analysis included the highest total serum bilirubin concentration (TsB), the lowest hemoglobin concentration (Hgb), and the highest direct bilirubin (DBil) and ferritin concentrations obtained during hospitalization.

Among the described group of newborns, patients were qualified for phototherapy and exchange transfusion (ET) based on charts developed by the American Academy of Pediatrics (AAP) for children born ≥35 weeks of gestation; for children born <35 weeks, gestation charts designed by Vinod K. Butani et al. were used [8,9].

In the authors’ center, intravenous immunoglobulin (IVIG) infusion was used when the bilirubin concentration was rising rapidly, typically when its level was 2–3 mg/dL below the threshold for ET. A rapid increase in TsB was defined as an increase exceeding more than 5 mg/dL per 24 h or 0.3 mg/dL per hour despite the use of phototherapy. This is a retrospective study without a previously planned strict procedure; therefore, decisions to use phototherapy or IVIG administration could depend on other factors such as the patient’s history (e.g., previous use of phototherapy, past IUT, antibodies’ type, and titer). According to reports from other centers, in cases of Rhesus hemolytic disease and newborns who have undergone IUT, phototherapy was usually initiated immediately after birth [10]. In newborns qualified for ET, IVIG infusion was used while awaiting the preparation of the ET procedure. This approach allowed us to withhold the ET procedure in several cases due to a decline in the bilirubin level.

Our study defined cholestasis as a DBil concentration exceeding 1 mg/dL, regardless of the TsB concentration [11].

The laboratory test results mentioned above and applied management were compared between newborns who received IUT (*n* = 46) and children who did not require such treatment (*n* = 228).

Qualitative variables were presented with absolute and relative numbers of observations. Numerical parameters were presented with basic descriptive statistics. The normality of the data distribution was assessed with the Shapiro–Wilk test, accompanied by skewness and kurtosis. Variance homogeneity verification was processed using the Levene test. Groups of children with and without IUT were compared with Student’s t-test or Mann–Whitney U test for numeric parameters and the Pearson Chi-square test or Fisher exact test for categorical parameters, as appropriate. All tests were performed with the assumption of statistical significance when *p* < 0.05. R software was used to produce the analysis outcomes, version R-4.1.2.

## 3. Results

### 3.1. Characteristics of the Study Group

Our study group consisted of *n* = 274 children, out of which 46.7% were female and 53.3% were male. The mean gestational age was 37.33 ± 1.85 weeks. Almost one in three children was born before 37 weeks of gestation.

The newborns’ average birth weight was 3159.41 ± 506.23 g. In total, 8.4% of the neonates were born with a low birth weight (LBW), defined as a birth weight below 2500 g. The birth weight percentiles ranged from 0.40 to 100.00, with a mean value of 63.20 ± 25.28. There were three neonates described as SGA, whose birth weight was under the 10th percentile.

Alloimmunization was most often applied to Rh (55.3%) and AB0 (36.3%) group antigens. In our study group, 16.8% of children required IUT. In the subgroup of children who experienced IUT [*n* = 46], the average number of procedures performed was 3.59 ± 2.54, with a minimum of 1.00 and maximum of 11.00 IUTs. Detailed characteristics of the analyzed parameters are presented in Table 1.

### 3.2. Laboratory Test Results and Management of Newborns with HDFN

In our study group, the mean Hgb concentration in the first measurement after birth was 15.62 g/dL, with the lowest concentration being 1.6 g/dL. Thirty-seven (13.5%) children required top-up transfusions, including 11 (4.01%) patients who received it more than once, with a maximum of three top-up transfusions.

In 231 (84.3%) newborns, the TsB concentration was measured in umbilical cord blood or right after birth in peripheral blood samples. The mean TsB concentration was 3.05 mg/dL, and the highest concentration measured right after birth was 9.9 mg/dL. The maximum concentration of TsB during hospitalization was 50.9 mg/dL; it should be noted that the DBil concentration in this patient reached 50.2 mg/dL. The highest TsB concentrations were observed on average on the 5th day of life.

Twelve (4.4%) newborns required ET, some of them more than once, with a maximum of six ETs performed in one patient. The average number of ETs performed in one neonate was 1.5. Moreover, 193 (70.4%) neonates received phototherapy for an average of 6.1 days. The most prolonged duration of phototherapy was 21 days. IVIG infusions were used in 64 (23.3%) patients, with four infusions being the highest number of procedures performed in one patient.

The serum ferritin concentration was measured in 110 (40.1%) children; in 102 (92.7%) newborns, it was determined with a measurable value, with a mean concentration of 688.68 ng/mL. The rest of the patients’ results exceeded the detection range for the ferritin measurement lab method (>1650 ng/mL).

During hospitalization, an elevated DBil concentration was found in 88 (32.1%) newborns from the study group. The DBil concentration measured right after birth ranged between 0.07 mg/dL and 5.51 mg/dL, with a mean concentration of 0.63 mg/dL. The highest DBil concentration reported during the hospital stay in one of the neonates reached 50.2 mg/dL.

The average length of stay was 8.39 days, while the most extended hospital stay lasted 56 days. In our study group, three (1.1%) patients died. The results are presented in Table 2.

### 3.3. Comparison of Children with and without Intrauterine Fetal Transfusions (IUT)

The study cohort of neonates diagnosed with HDFN included children who underwent IUT and those who did not require such intervention. The laboratory test results and management applied during the data analysis were compared between both groups. Forty-six neonates from the study cohort received at least one IUT. This intervention was not performed in 228 children included in this study.

The results differed remarkably between the two groups. Hgb concentrations were significantly lower in children who required IUT compared to children in whom IUT was not performed, both measured immediately after birth and in the following days (the lowest Hgb concentration during hospitalization); the mean difference in the Hgb concentration in both groups was MD = −3.30 (CI95 −4.10; −2.20, *p* < 0.001) and MD = −4.15 (CI95 −5.20; −3.10, *p* < 0.001) for these measurements, respectively. The number of children who required a top-up transfusion during hospitalization in the neonatal unit was significantly higher in the group treated prenatally with IUT (32.6% vs. 9.2%), *p* < 0.001.

The TsB concentration was measured in cord blood or peripheral blood samples right after birth in 100.0% of children who underwent IUT and in 81.1% of newborns without IUT, *p* = 0.003, as in some of these patients, the diagnosis of HDFN was made in the following hours or days of life. The TsB concentrations measured in cord blood or right after birth were higher in the group of patients who received IUT compared to the group of newborns for whom such intervention in the prenatal period was not necessary; the mean difference in the TsB concentrations was 2.43 mg/dL (CI95 1.48; 2.68), *p* < 0.001. Both groups did not differ significantly regarding the median value of the highest TsB concentration during hospitalization (10.53 vs. 11.73 mg/dL, *p* = 0.532), which was observed on average on the 4th day of life. The number of children who required ET was significantly higher in the group of newborns after IUT (17.4%, *n* = 8 vs. 1.8%, *n* = 4), *p* < 0.001, as was the number of children who received phototherapy (97.8% vs. 64.9%), *p* < 0.001. Newborns who underwent IUT also more often required IVIG infusion (67.4% vs. 14.5%), *p* < 0.001.

The ferritin concentration was measured in 87.0% of newborns treated with IUT and in 30.7% of children who did not require this intervention, *p* < 0.001. A significant difference was observed in the number of children with the highest ferritin concentrations, i.e., exceeding the detection range for the ferritin measurement method used (>1650 ng/mL). In the group of newborns treated with IUT, a ferritin concentration exceeding 1650 ng/mL was observed in eight neonates (20.0%), while among the neonates who did not require IUT prenatally, none of the children had such high ferritin levels, *p* < 0.001. In patients with a ferritin concentration <1650 ng/mL (measurable value, not exceeding the detection range for the used measurement method), the results differed significantly between the groups, with higher ferritin concentrations observed in the group of neonates after IUT with a mean difference of 563.302 ng/mL (CI95 454.56; 672.04), *p* < 0.001.

Our analysis found a higher risk of cholestasis in the group of newborns requiring IUT (52.2% vs. 28.1%, *p* = 0.03). We also observed a longer length of hospital stay in the group of patients who underwent IUT; the mean difference in the length of stay was 3.00 days (CI95 2.00; 5.00), *p*< 0.001. The results are presented in Table 3.

## 4. Discussion

Specific antibodies against fetal red blood cells formed in the mother’s bloodstream cause HDFN. The disease may be provoked by a blood group antigen inherited by the child from its father. This may involve Rhesus antigens (D, d, C, c, E, e), as well as other blood group systems such as Kidd (Jka, Jkb), Kell (K, k), MNS (M, N, S, s), Duffy (Fya, Fyb), Lewis (Lea, Leb), AB0 and others [12]. In our study group, most cases of alloimmunization involved Rhesus (55.1%) and AB0 (36.1%) blood group system antigens. Thirteen (4.7%) newborns had antibodies against various blood group antigens. Specific antibodies are formed when fetal blood cells enter the mother’s bloodstream. Subsequently, these antibodies cross the placenta and coat fetal erythrocytes, causing hemolysis. Bilirubin, produced from the breakdown of fetal red blood cells, does not threaten the fetus as it is excreted through the placenta [13].

Despite the prophylactic administration of immunoglobulin to prevent the immunization of Rh(D)-negative pregnant women, there are still neonates born with hemolytic disease, requiring treatment and observation. Our research represents the first Polish study to cover the topic of HDFN in such a large cohort of newborns.

Depending on the severity of the red blood cell breakdown process, fetal anemia may lead to circulatory failure, hydrops fetalis, and even intrauterine fetal demise [13,14,15]. To assess the severity of symptoms in fetal anemia, an ultrasound examination with the assessment of the MCA flow is performed. In fetal anemia, increased blood flow is observed in all blood vessels secondary to reduced blood viscosity, resulting in increased cardiac output. To determine the indications for IUT, MoM is estimated for the MCA-PSV value; the indication for IUT is a MoM value exceeding 1.5 [14,15,16]. In our study group, 46 newborns were qualified for IUT and some of them required this procedure multiple times, with a maximum of 11 IUTs.

In the case of severe hemolysis, an increased concentration of TsB can be detected in the umbilical cord blood. In the analyzed group, the average TsB concentration measured in the cord blood or right after birth in a peripheral blood sample was 3.05 mg/dL, the maximum was 9.9 mg/dL, and higher concentrations were observed in children who required IUT. After birth, the newborn’s liver is unable to excrete the large amounts of bilirubin produced due to the immaturity of the neonate’s enzyme system. This causes a rapid increase in the TsB concentration, which may result in kernicterus and cerebral palsy [13,17]. Each newborn with HDFN requires careful monitoring to reduce the risk of severe consequences by early treatment initiation. According to some studies, lower mean maximum TsB concentrations were observed in the group of children after IUT [18]. However, our research does not confirm this, as the highest TsB concentrations were similar in both groups and the differences were not statistically significant.

The most common treatment for hyperbilirubinemia is phototherapy or, in more severe cases, an ET [13,19]. According to reports, in newborns with Rhesus hemolytic disease, the duration of phototherapy is shorter in children who undergo IUT [10,18]. The ET procedure carries the risk of complications such as thrombocytopenia, hypocalcemia, hyperkalemia, leukopenia, and sepsis [17]. Cardiac arrhythmias, cardiac arrest, apnea, necrotizing enterocolitis, and death may also occur [13,19]. The jeopardy of serious complications is estimated at 12% [20]. The neonate’s risk of death following ET is 8%, and according to another source, 0.3% for full-term newborns and about 10% for prematurely born children [13,19]. Due to its possible severe consequences, qualification for the procedure should be carefully deliberated. Indications are not always clear, especially on the first day of a newborn’s life. In addition to the TsB concentration, various other factors need to be taken into consideration, including its rate of increase, the child’s gestational age, the presence of possible neurological symptoms, as well as hyperbilirubinemia neurotoxicity risk factors, counting albumin concentration, sepsis, and significant clinical instability in the previous 24 h [20]. In neonates with Rhesus hemolytic disease, a similar risk of requiring ET is reported in both newborns with past IUT and those without. However, the authors note that specific criteria for early ET may influence the results [10,18].

In some centers, IVIG infusions are used to treat hyperbilirubinemia caused by HDFN [8,13,19]. Several randomized studies did not confirm IVIG administration’s impact on the frequency of ET [18]. However, the AAP guidelines suggest that IVIG is administered to neonates with isoimmune hemolytic disease whose TsB reaches or exceeds the escalation of care threshold [20]. In the described group, most children (70.4%) received phototherapy, and 23.3% received IVIG infusion. Despite such treatment, some children (4.1%) still received the ET procedure; in each case, the procedure was carried out without any significant complications. Other methods of treating hyperbilirubinemia have been described, e.g., albumins, phenobarbital, prebiotics, zinc, and metalloporphyrins, but we do not have sufficient data to consider them effective [13].

In our study group, newborns who required IUTs were more likely to need treatment with both phototherapy and ET. IVIG infusions were also used more often. Unlike in the literature, the length of phototherapy did not differ in our patients with and without IUT [10]. However, it should be noted that our study includes various types of red cell immunization, not only Rhesus hemolytic disease. In contrast to the literature, our study found a difference in the frequency of ET between the two groups [18].

In children with HDFN, apart from hyperbilirubinemia, anemia is a significant problem and as many as 68–83% of newborns require top-up transfusions [13]. In our study group, this number was smaller, but we analyzed only the period of stay in the neonatal unit, and transfusions performed later in life were not considered. The diminished number of ETs described in recent years is associated with an increased need to perform top-up transfusions [13]. Erythropoietin, folic acid, iron, and vitamin E are also used to treat anemia. Research on the effectiveness of these therapies in children with anemia due to HDFN is still ongoing [13]. It is noteworthy that further treatment or the discontinuation of iron supplementation should be considered depending on ferritin levels [13]. According to the literature, more newborns require top-up transfusions after IUT compared to those who do not undergo this procedure [10,18].

In the described group, lower Hgb concentrations measured on the first day of life, as well as the lowest concentrations determined during the entire hospitalization, were found in the group of children who had undergone IUT. In our study, newborns who received IUT prenatally required top-up transfusions more often than those who did not undergo prenatal interventions.

Another problem affecting the described group of patients is an increased iron concentration due to hemolysis and top-up transfusions, both intrauterine and after birth [13]. The iron overload may cause liver and heart damage, and it is responsible for increased susceptibility to infections [13,21]. The literature describes several cases of children who required chelation therapy due to very high ferritin levels, causing liver failure [22,23]. The authors of this study have already published a paper research on the use of chelation therapy in iron overload due to HDFN [24]. In newborns with HDFN, higher ferritin concentrations were observed in patients who underwent IUT. Very high ferritin concentrations (exceeding the detection range for the test) were also more frequently reported in this group.

Some newborns with HDFN also have an increased DBil concentration [13,25,26,27]. This problem affects approximately 7–13% of children born from pregnancies complicated by alloimmunization. It should be taken into consideration that, in the cited literature, the authors diagnosed cholestasis according to now outdated criteria, i.e., a DBil concentration above 2 mg/dL, and not above 1 mg/dL according to the new guidelines; this would have undoubtedly affected the diagnosis statistics [11]. In our study group, cholestasis was found in 32.1% of newborns. According to the literature, the risk of cholestasis is higher in newborns who require IUT and in those children whose top-up transfusions performed after birth were necessitated by anemia in the course of Rhesus alloimmunization [13,25]. We also found a higher incidence of cholestasis in children after IUT in our study group.

Despite an extensive literature review, we could not find a case report of a patient with a DBil concentration as high as that in several neonates described in our study group; therefore, we described this case in a separate article [28]. The highest concentration of DBil that we were able to find in the published research was 31.6 mg/dL, with a total bilirubin concentration of 45.2 mg/dL in a full-term child with kernicterus due to hyperbilirubinemia caused by Rhesus alloimmunization [29,30].

## 5. Study Limitations

1. We obtained the data retrospectively, so there was no previously established HDFN management protocol; therapeutic decisions were made based on the doctor’s knowledge and individual experience, and not on previously established guidelines. This also applies to the doctor’s preferences regarding the first Hgb concentration measurement in the umbilical cord or peripheral blood sample.
2. Furthermore, the patients’ highest ferritin concentrations remained unknown due to laboratory limitations, as the test’s detection range did not exceed >1650 ng/mL.
3. In some neonates, the diagnosis of HDFN (especially in the case of AB0-alloimmunization) was made a few days after birth. Therefore, there is no complete data on the hemoglobin and bilirubin concentrations measured in cord or peripheral blood samples right after birth.

## 6. Conclusions

HDFN is a still common disease that may be challenging for obstetricians, neonatologists, and pediatricians.

Alloimmunization with subsequent fetal anemia requiring IUT is associated with a higher risk of complications in the neonatal period such as severe hyperbilirubinemia, cholestasis, iron overload, and anemia. Those patients more often require additional treatment with phototherapy, IVIG infusions, as well as exchange and top-up transfusions, compared to neonates with HDFN not requiring intrauterine therapy. Therefore, they need more thorough observation and more extended hospitalization in the neonatal period.

The careful management of patients with HDFN and early treatment are necessary to avoid major complications, especially in the children who receive IUT.

## Figures and Tables

**Table 1:** Characteristics of the study group (*n* = 274).

Variable	*n* (%)	M	SD	Me	Min	Max
Sex						
Female	128 (46.7)	-	-	-	-	-
Male	146 (53.3)	-	-	-	-	-
Gestational age [weeks]	-	37.33	1.85	38.00	28.00	41.00
Gestational age < 37 weeks	84 (30.7)	-	-	-	-	-
Birth weight [g]	-	3159.41	506.23	3190.00	1400.00	4570.00
Birth weight < 2500 g	23 (8.4)	-	-	-	-	-
Birth weight percentile	-	63.20	25.28	66.50	0.40	100.00
Birth weight percentile < 10	3 (1.1)	-	-	-	-	-
Types of red cell alloimmunization						
Rh	151 (55.1)	-	-	-	-	-
Kidd	0 (0.0)	-	-	-	-	-
Kell	8 (2.9)	-	-	-	-	-
Duffy	1 (0.4)	-	-	-	-	-
MNs	2 (0.7)	-	-	-	-	-
AB0	99 (36.1)	-	-	-	-	-
Rh/Duffy	5 (1.8)	-	-	-	-	-
Rh/Duffy/Kidd	1 (0.4)	-	-	-	-	-
Rh/Kell	1 (0.4)	-	-	-	-	-
Rh/Kidd	5 (1.8)	-	-	-	-	-
Rh/MNs	1 (0.4)	-	-	-	-	-
Number of IUT-treated neonates	46 (16.8)	-	-	-	-	-
Number of IUTs in IUT-treated neonates	-	3.59	2.54	3.00	1.00	11.00

IUT—intrauterine transfusion, M—mean, SD—standard deviation, Me—median, Min—minimal value, Max—maximal value.

**Table 2:** Laboratory test results and management of newborns with HDFN (*n* = 274).

Variable	*n* (%)	M	SD	Me	Min	Max
Hgb concentration [g/dL]						
Hgb after birth	-	15.62	3.33	16.00	1.60	23.30
Howest Hgb during the hospital stay	-	14.06	3.86	14.60	1.60	23.10
Number of TU-treated neonates	37 (13.5)	-	-	-	-	-
Number of TUs in TU-treated neonates	-	1.33	0.59	1.00	1.00	3.00
Number of neonates with measured TsB ^1^	231 (84.3)	-	-	-	-	-
TsB concentration [mg/dL]						
TsB after birth	-	3.05	1.77	2.44	0.30	9.90
Highest TsB during the hospital stay	-	11.85	4.92	11.66	2.12	50.90
Day after birth with the highest TsB	-	4.60	2.29	4.00	1.00	18.00
Number of ET-treated neonates	12 (4.4)	-	-	-	-	-
Number of ETs in ET-treated neonates	-	1.50	1.45	1.00	1.00	6.00
Number of PT-treated neonates	193 (70.4)	-	-	-	-	-
Duration of PT in PT-treated neonates [days]	-	6.10	3.88	5.00	1.00	21.00
Number of IVIG-treated neonates	64 (23.4)	-	-	-	-	-
Number of IVIG infusions in IVIG-treated neonates	-	1.50	0.78	1.00	1.00	4.00
Number of neonates with measured ferritin concentration	110 (40.1)	-	-	-	-	-
Number of neonates with ferritin < 1650 ng/mL ^2^	102 (92.7)	-	-	-	-	-
Number of neonates with ferritin > 1650 ng/mL ^2^	8 (7.3)	-	-	-	-	-
Ferritin concentration ^3^ [ng/mL]	-	688.68	366.49	622.10	117.20	1617.60
Number of neonates with DBil > 1.0 mg/dL	88 (32.1)	-	-	-	-	-
DBil concentration [mg/dL]						
DBil after birth	-	0.63	0.64	0.44	0.07	5.51
Highest DBil during the hospital stay	-	2.56	6.15	1.42	1.01	50.20
Length of stay [days]	-	8.39	7.08	6.00	1.00	56.00
Number of deaths	3 (1.1)	-	-	-	-	-

Hgb—hemoglobin, TU—top-up transfusion, TsB—total serum bilirubin, ET—exchange transfusion, PT—phototherapy, IVIG—intravenous immunoglobulin, DBil—direct bilirubin concentration, M—mean, SD—standard deviation, Me—median, Min—minimal value, Max—maximal value. ^1^ TsB measured in cord blood or peripheral blood samples right after birth. ^2^ Base for % calculation was *n* = 110 (all children with measured ferritin concentration). ^3^ Base for calculations was *n* = 102 (all children with ferritin concentration <1650 ng/mL). Ferritin > 1650 ng/dL—values above the detection range for the ferritin measurement method.

**Table 3:** Comparison of children with and without IUT.

Variable	Children with IUT (%, *n* = 46)	Children without IUT (%, *n* = 228)	MD (95% CI)	*p*
Hgb concentration [g/dL]				
Hgb after birth, median (IQR)	13.05 (11.48; 14.97)	16.35 (14.67; 17.95)	−3.30 (−4.10; −2.20)	**<0.001**
lowest Hgb, median (IQR)	11.15 (8.43; 12.97)	15.30 (12.70; 17.20)	−4.15 (−5.20; −3.10)	**<0.001**
Number of TU-treated neonates	15 (32.6)	22 (9.6)	-	**<0.001** **
Number of neonates with TsB ^1^ measured after birth	46 (100.0)	185 (81.1)	-	**0.003** ***
TsB concentration [mg/dL]				
TsB after birth, median (IQR)	4.61 (3.08; 5.88)	2.18 (1.71; 3.23)	2.43 (1.48; 2.68)	**<0.001**
highest TsB, median (IQR)	10.53 (8.46; 14.61)	11.73 (8.94; 14.38)	−1.20 (−1.85; 1.06)	0.532
Day after birth with the highest TsB, median (IQR)	4.00 (3.00; 5.75)	4.00 (3.00; 5.00)	0.00 (−1.00; 1.00)	0.896
Number of ET-treated neonates	8 (17.4)	4 (1.8)	-	**<0.001** ***
Number of PT-treated neonates	45 (97.8)	148 (64.9)	-	**<0.001** **
Duration of PT [days], median (IQR); (PT-treated neonates)	6.00 (4.00; 8.00)	4.00 (3.00; 9.00)	2.00 (0.00; 2.00)	0.128
Number of IVIG-treated neonates	31 (67.4)	33 (14.5)	-	**<0.001** **
Number of neonates with measured ferritin concentration	40 (87.0)	70 (30.7)	-	**<0.001** **
Number of neonates with ferritin >1650 ng/mL ^2^	8 (20.0)	0 (0.0)	-	**<0.001** ***
Number of neonates with ferritin <1650 ng/mL ^3^	32 (80.0)	70 (100.0)	-	**<0.001** ***
Ferritin concentration ^4^ [ng/mL], M ± SD	1075.26 ± 270.75	511.96 ± 250.36	563.30 (454.56; 672.04)	**<0.001** *
Number of children with cholestasis	24 (52.2)	64 (28.1)		**0003**
Length of stay [days], median (IQR)	9.00 (7.00; 14.00)	6.00 (4.00; 9.00)	3.00 (2.00; 5.00)	**<0.001**
Number of deaths	1 (2.2)	2 (0.9)	-	0.425 ***

Hgb—hemoglobin, TU—top-up transfusion, TsB—total serum bilirubin, ET—exchange transfusion, PT—phototherapy, IVIG—intravenous immunoglobulin, DBil—direct bilirubin concentration, M—mean, SD—standard deviation, IQR—interquartile range, MD—mean or median difference (children with IUT vs. children without IUT), CI—confidence interval. Groups compared with Student’s *t*-test for independent groups * or Mann–Whitney U test (numeric variables) and Chi-square Pearson test ** or exact Fisher test *** (nominal variables). ^1^ TsB measured in cord blood or peripheral blood sample right after birth. ^2^ Ferritin >1650 ng/dL—values above the detection range for used ferritin measurement method. The base for % calculation was *n* = 110 (all children with measured ferritin concentration). ^3^ Highest ferritin concentration marked with a measurable value (<1650 ng/dL—results within the detection range of the test). The base for % calculation was *n* = 110 (all children with measured ferritin concentration). ^4^ The base for calculation was the results obtained in children with ferritin concentration <1650 ng/dL. Statistically significant results are highlighted in bold.

## Data Availability

The raw data supporting the conclusions of this article will be made available by the authors on request.
